# Preeclampsia and toxic metals: a case-control study in Kinshasa, DR Congo

**DOI:** 10.1186/s12940-016-0132-1

**Published:** 2016-04-05

**Authors:** Jean-Pierre Elongi Moyene, Hans Scheers, Barthélémy Tandu-Umba, Vincent Haufroid, Baudouin Buassa-bu-Tsumbu, Fons Verdonck, Bernard Spitz, Benoit Nemery

**Affiliations:** Department of Gynecology and Obstetrics, University of Kinshasa, and General Hospital of Kinshasa, Kinshasa, Democratic Republic of Congo; Department of Public Health and Primary Care, Centre for Environment and Health, KU Leuven, Leuven, Belgium; Louvain centre for Toxicology and Applied Pharmacology, Institut de recherche expérimentale et clinique, Université catholique de Louvain, Brussels, Belgium; Faculty of Medicine, KU Leuven, Leuven, Belgium; Department of Development and Regeneration (Pregnancy, Foetus and Newborn), KU Leuven, Leuven, Belgium; Division of Gynecology and Obstetrics, UZ Leuven, Leuven, Belgium; Hôpital Général de Kinshasa, Avenue de l’Hôpital, Commune de la Gombe, Kinshasa, DR Congo

**Keywords:** Metal pollution, Lead, Preeclampsia, Hypertension, Seasonality, Developing country, Global health

## Abstract

**Background:**

Preeclampsia is frequent in Kinshasa (Democratic Republic of Congo), especially during the dry season. We tested whether preeclampsia was associated with exposure to environmental metals.

**Methods:**

Using a case-control design, 88 women hospitalized with preeclampsia (cases) and 88 healthy pregnant women from the antenatal clinic (controls) were included in the study; 67 and 109 women were enrolled during the rainy and dry season, respectively. The concentrations of 24 elements were quantified by inductively coupled plasma mass spectrometry (ICP-MS) in 24-h urine collections. Differences in the urinary excretion of metals were investigated between cases and controls, and the interaction with season was assessed.

**Results:**

Cases and controls were well matched regarding age, parity and duration of pregnancy. In controls, the urinary concentrations of most elements were substantially higher than reference values for adults from industrially developed countries, e.g. for lead: geometric mean (GM) 8.0 μg/L [25^th^-75^th^ percentile 3.1–13.8]. The daily urinary excretions of 14 metals were significantly higher in women with preeclampsia than in control women, e.g. for lead: GM 61 μg/day (25^th^–75^th^ percentile 8–345) in women with preeclampsia *vs* 9 μg/day (25^th^–75^th^ percentile 3–21) in controls (*p* < 0 · 001). A significant interaction was found between season and preeclampsia for several elements, with higher urinary excretions in preeclamptic women than controls during the dry season, but not during the rainy season.

**Conclusions:**

This study revealed not only that women with preeclampsia excrete higher amounts of several toxic metals, especially lead, than control women, but also that this excretion exhibits seasonal variation, thus possibly explaining the high incidence and seasonal variation of preeclampsia in Kinshasa. Although the exact sources of this exposure are unknown, these findings underscore the need for preventing environmental exposures to lead and other toxic metals.

**Electronic supplementary material:**

The online version of this article (doi:10.1186/s12940-016-0132-1) contains supplementary material, which is available to authorized users.

## Background

Preeclampsia is a leading cause of maternal and perinatal deaths, especially in poor countries [1]. With almost 10 million inhabitants, Kinshasa, the capital of the Democratic Republic of Congo, is the second most populated urban area of sub-Saharan Africa. Not only is the incidence of preeclampsia high in Kinshasa, its frequency also exhibits a striking seasonal variation: a retrospective study of more than 17,500 pregnancies between 2003 and 2007 showed prevalences of preeclampsia of 6 % during the rainy season and 13 % in the dry season [2].

The causes for the high incidence and seasonal variation of preeclampsia in Kinshasa are not known. Since a low intake of dietary anti-oxidants contributes to preeclampsia [3], it is possible that the risk of preeclampsia increases in the dry season because of a lower availability of fresh vegetables. Although some evidence supports the latter mechanism [4], another complementary explanation is that exposure to pro-oxidant metals plays a role. Preeclampsia has been associated with exposure to metals, such as lead [5], and a high degree of pollution by trace metals has been shown in Kinshasa [6]. The extent and sources of this pollution are not well known, but like many other megacities in low-to-middle income countries, Kinshasa suffers from considerable air, soil and water pollution, as a result of heavy traffic (with many old cars and resuspension of road dust), smoke from burning biomass and refuse, road and building works, unregulated activity of lots of small enterprises and workshops (welding, battery recycling, …) in residential areas, etc. Studies in US children have documented that the uptake of lead increases during the dry season because of resuspension of lead-contaminated soil dust [7].

The objectives of the present case-control study were to determine whether women with preeclampsia had evidence of a higher exposure to trace metals than control pregnant women, and whether this was more the case in the dry season than in the wet season.

## Methods

### Study design and setting

In a case-control study design, we compared the 24-h urinary excretion of metals and metalloids in pregnant women suffering from preeclampsia or eclampsia (cases) with that of healthy pregnant women (controls).

The study was conducted at the General Hospital of Kinshasa, the largest hospital in the Democratic Republic of Congo. Data were collected during two periods with different meteorological characteristics: the rainy season (inclusions between 1 March and 18 April 2011) and the dry season (inclusions between 1 July and 2 September 2011).

### Participants

All participants were pregnant women having had at least one prior pregnancy (i.e. nulliparous women were not included). Women with preeclampsia were inpatients recruited from the hospital’s obstetric ward or intensive care unit. Preeclampsia was defined, according to current diagnostic criteria of the National High Blood Pressure Education Program [8], as the occurrence, in the second or third trimester of pregnancy, of hypertension (systolic blood pressure > 140 mm Hg and diastolic blood pressure > 90 mm Hg after at least 15 min of rest), combined with proteinuria (positive dipstick test or > 300 mg proteins/24 h), with or without oedema. Eclampsia was defined as the occurrence of seizures in a pregnant woman presenting the above criteria, in the absence of neurological disease or brain injury. Concurrent control subjects were selected from the outpatient antenatal care unit of the same hospital so as to match patients in terms of age, gestational age, type of pregnancy (single or multiple), and number of live-born children. Subjects with chronic and debilitating disease, smokers, and regular consumers of alcohol were not included. All selected subjects were informed about the purpose and procedures of the study. Participation in the study was voluntary and there were no refusals to participate among eligible patients with pre-eclampsia. Controls were offered 1,000 francs (about 1 US dollar) to cover transport costs and only few refused to participate: four control women declined to participate because they found 1,000 francs insufficient to cover their travel expenses; two control women who had accepted and received 1,000 francs did not report back. These controls were replaced by other women.

The final protocol was approved by the ethical committee of the National Order of Physicians of the DR Congo (COM/013/HPGRK/2011).

### Variables

Medical history (including hypertension and diabetes) and obstetrical history were obtained from the patient’s clinical notes. Height and weight were measured to calculate body mass index (BMI). A questionnaire, elaborated in house and consisting of simple questions in French or Lingala, was administered face-to-face by various interviewers to cases and controls to assess educational level (illiterate; elementary or secondary school; higher education), annual income (low, intermediate or high), occupation (paid work outside the home or not), cooking mode (biomass fuel, gas, electricity), geophagy (the consumption of clay, a frequent habit among pregnant women in Africa [9]) and specific activities with possible metal pollution close to the residence (recycling of batteries, spray painting, or welding). Area of residence was registered as one of the 24 “communes” in the Kinshasa agglomeration.

In hospitalized patients with preeclampsia, urine was collected by vesical catheterization, starting at admission; the catheter was inserted without use of a lubricating agent and 24 h urine was collected in a sterile graduated plastic container. Control subjects received a plastic vessel (with a large opening and closely fitting lid) and they were instructed to void their urine directly into the vessel without external contamination during a set period of 24 h, and to bring the containers back to the hospital as soon as the collection was finished.

After recording total volume, approximately 20 mL urine were transferred into polystyrene containers with screw caps (Plastiques-Gosselin, Hazebrouck, France), which were kept frozen and then transported (in three batches) in isothermal boxes to Belgium by commercial flights.

### Measurements

As in previous publications [10, 11], the urinary concentrations of 24 metals and metalloids [lithium (Li), beryllium (Be), aluminium (Al), vanadium (V), chromium (Cr), manganese (Mn), cobalt (Co), nickel (Ni), copper (Cu), zinc (Zn), arsenic (As), selenium (Se), molybdenum (Mo), cadmium (Cd), indium (In), tin (Sn), antimony (Sb), tellurium (Te), barium (Ba), platinum (Pt), thallium (Tl), lead (Pb), bismuth (Bi), and uranium (U), all called “metals” hereafter] were simultaneously measured by inductively coupled argon plasma mass spectrometry (ICP-MS), using validated and ISO15189 certified procedures, with quality control/quality assurance procedures, as described in the additional files. Four metals (Be, In, Pt, and Bi) were not considered further because more than half of the samples had concentrations below the limit of detection (LOD). Creatinine was determined by a modified Jaffe reaction using an Olympus AU2700 analyzer (Olympus, Hamburg, Germany).

### Data presentation and statistical analysis

**Fig. 1:**
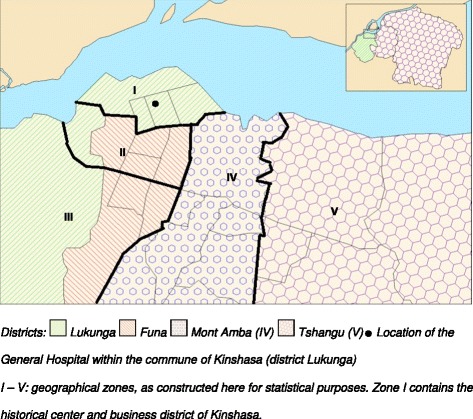
Map of Kinshasa with administrative entities and location of the General Hospital. I – V: geographical zones, as constructed here for statistical purposes. Zone I contains the historical center and business district of Kinshasa

Data management and statistical analysis were performed in SAS 9.3 (SAS Institute, Cary, USA). Except for Be, In, Pt and Bi, only few measurements were below the LOD; these values were assigned a value at half the LOD. Urinary metal concentrations (μg/L) were multiplied by the daily urine volume (L/day) to obtain the total amount of metal excreted per 24 h (μg/day). Metal concentrations (μg/L) and daily metal excretions (μg/day) were not normally distributed, but all sets of values met the assumption of normal distribution after log-transformation. Data are reported as geometric means with 25^th^ and 75^th^ percentiles. Two-way ANOVA, followed by Tukey’s post hoc tests, were performed to disentangle the separate and joint effects of preeclampsia and season. Categorical variables were evaluated by chi square tests or Fisher exact tests, and interpreted as odds ratios (OR) with 95 % confidence intervals (CI). To compare the geographical distribution between cases and controls, communes were pooled in five zones, largely corresponding with the four districts of Kinshasa (Fig. 1). A correlation analysis between the metal excretion values was performed and this was followed by a principal component analysis (PCA) to derive a composite metal excretion variable. The significance level was set at *p* < 0.05. The Benjamini-Hochberg method [12] to reduce the risk of type I errors when making multiple comparisons, was used but this did not modify the conclusions obtained without applying such correction, and the *p* values shown are those obtained without correcting for multiple testing.

## Results

A total of 178 pregnant women were enrolled, equally divided among the preeclamptic and control groups. For one control woman and one preeclamptic woman, no urine data were available due to damaged containers. Thus, data from 88 women with preeclampsia (34 included during the rainy season and 54 during the dry season) and 88 controls (33 included during the rainy season and 55 during the dry season) were available for analysis.

The matching strategy led to the case and control groups being highly similar except, of course, for blood pressure (Table 1). In each group, seven women had a twin pregnancy. In regard to environmental factors (Table 2), cases and controls did not differ with regard to residential location, cooking mode and prevalence of geophagy. However, artisanal activities close to home (such as recycling of batteries, spray painting and welding) tended to be more frequently reported in the preeclamptic group (*p* = 0.064). Pooling these activities into a single category gave a significant association with preeclampsia (OR = 2.34, 95 % CI 1.13–4.85, *p* = 0.02).

**Table 1:** Personal characteristics of women with or without preeclampsia

	Control (*N* = 88)	Preeclamptic (*N* = 88)
Age (years)	26 · 7 ± 5 · 9	27 · 1 ± 6 · 1
Height (m)	1 · 62 ± 0 · 07	1 · 62 ± 0 · 09
Weight (kg)	73 · 5 ± 8 · 8	73 · 6 ± 9 · 8
BMI (kg/m^2^)	27 · 9 ± 3 · 0	28 · 1 ± 3 · 7
Blood pressure (mm Hg)		
Systolic	112 ± 12	186 ± 25
Diastolic	72 ± 10	119 ± 23
Gestational age (weeks)	36 · 2 ± 2 · 2	36 · 8 ± 2 · 1
Parity		
1	36 (41 %)	44 (50 %)
2	21 (24 %)	18 (20 %)
3	19 (22 %)	15 (17 %)
3+	12 (14 %)	11 (13 %)
Education level		
1 illiterate	2 (2 %)	4 (5 %)
2 elementary school	75 (85 %)	75 (85 %)
3 secondary school	11 (13 %)	9 (10 %)

Data are arithmetic means ± SD, or counts (%)

**Table 2:** Environmental characteristics of women with or without preeclampsia

	Control (*N* = 88)	Preeclamptic (*N* = 88)	*P* ^#^
Residence (zone)^a^			0 · 22
I	20 (23 %)	16 (18 %)	
II	17 (20 %)	15 (17 %)	
III	11 (13 %)	14 (16 %)	
IV	29 (33 %)	22 (25 %)	
V	10 (11 %)	21 (24 %)	
Occupation			0 · 049
0 no paid work	33 (38 %)	46 (52 %)	
1 paid work	55 (62 %)	42 (48 %)	
Reported cooking mode			0 · 13
1 biomass fuel	13 (15 %)	21 (24 %)	
2 gas or electricity	75 (85 %)	67 (76 %)	
Reported geophagy			0 · 62
0 no	64 (73 %)	60 (68 %)	
1 yes	24 (27 %)	28 (32 %)	
Reported activities in vicinity			
spray painting	1 (1 %)	7 (8 %)	0 · 06
welding	5 (6 %)	8 (9 %)	
battery recycling	8 (9 %)	12 (14 %)	*0 · 02* ^†^
none of these	74 (84 %)	61 (69 %)	

Data are counts (%)

^#^ *P*-values for chi square or Fisher exact tests on frequencies. ^a^Zones as defined in Fig. 1. ^†^ *P*-value for an additional chi square test with spray painting, welding and battery recycling pooled vs no activities

A large proportion of samples collected in the dry season turned out to have unreliably low concentrations of creatinine (<0.3 g/L), probably because one batch had remained unfrozen during transportation or before.

Detailed figures for the metal concentrations found in the control women are given for reference in additional files (Additional file 1: Tables S1–S3). For many metals, the values were substantially higher than reference values found in other populations of non-pregnant adults from the USA [13, 14] or Belgium [15]. Urinary concentrations of all metals, except Li, were significantly higher in preeclamptic subjects than in controls (Table 3). The highest contrasts between the two groups were observed for Zn, Sn and Pb with approximately 9-fold differences in geometric means.

**Table 3:** Urinary metal concentrations (in μg/L) in pregnant women with or without preeclampsia in Kinshasa

	Control (*N* = 88)	Preeclamptic (*N* = 88)	Fold difference	*P* ^#^	Upper reference limit NHANES^†^	Upper reference limit Belgium^‡^
Li	6 · 30 (3 · 34-11 · 8)	7 · 02 (4 · 77-9 · 53)	1 · 1	0 · 30		100
Al	55 · 4 (21 · 9-172)	132 (48 · 5-400)	2 · 4	<0 · 001	34 · 0^b^	15
V	1 · 44 (0 · 97-1 · 90)	2 · 09 (1 · 21-3 · 49)	1 · 5	<0 · 001		1 · 5
Cr	0 · 88 (0 · 39-2 · 46)	4 · 57 (1 · 02-24 · 3)	5 · 2	<0 · 001	0 · 48^a^, 3 · 5^b^	0 · 55
Mn	9 · 20 (2 · 06-27 · 7)	44 · 5 (5 · 32-273)	4 · 8	<0 · 001	2 · 71^a^	0 · 75
Co	0 · 54 (0 · 21-1 · 37)	2 · 07 (0 · 75-4 · 19)	3 · 9	<0 · 001	4 · 69^a^, 1 · 8^b^	1 · 8
Ni	4 · 14 (2 · 42-8 · 98)	13 · 8 (6 · 3-25 · 1)	3 · 3	<0 · 001	12 · 0^b^	6
Cu	34 · 4 (10 · 6-134)	226 (69.5-562)	6 · 6	<0 · 001	55 · 0^b^	27
Zn	627 (186-1,129)	5,863 (1,229-40,950)	9 · 3	<0 · 001	766 · 8^b^	1620
As	26 · 8 (13 · 4-51 · 6)	46 · 9 (26 · 1-79 · 9)	1 · 7	<0 · 001	52 · 2^b^	300
Se	27 · 2 (13 · 5-54 · 3)	44 · 6 (24 · 0-71 · 8)	1 · 6	<0 · 001	182 · 0^b^	80
Mo	13 · 3 (7 · 2-29 · 8)	19 · 2 (10 · 7-35 · 6)	1 · 4	0.023	128 · 0^a^, 66 · 9^b^	150
Cd	0 · 53 (0 · 29-0 · 68)	1 · 78 (0 · 71-3 · 85)	3 · 3	<0 · 001	1 · 0^b^	1 · 5
Sn	1 · 25 (0 · 43-2 · 60)	10 · 8 (2 · 2-48 · 6)	8 · 7	<0 · 001	34 · 9^b^	4
Sb	0 · 46 (0 · 14-1 · 57)	1 · 95 (0 · 84-4 · 85)	4 · 2	<0 · 001	4 · 17^a^, <LOD^b^	0 · 35
Te	0 · 11 (0 · 07-0 · 19)	0 · 23 (0 · 12-0 · 26)	2 · 1	<0 · 001	<LOD^b^	0 · 4
Ba	11 · 7 (5 · 5-24 · 9)	34 · 8 (8 · 4-123 · 8)	3 · 0	<0 · 001	5 · 27^a^, 7 · 0^b^	9
Tl	0 · 23 (0 · 15-0 · 45)	0 · 36 (0 · 24-0 · 57)	1 · 6	<0 · 001	1 · 18^a^, <LOD^b^	0 · 6
Pb	7 · 98 (3 · 14-13 · 8)	71 · 5 (8 · 89 -398)	9 · 0	<0 · 001	4 · 93^a^, 4 · 0^b^	4
U	0 · 04 (0 · 02-0 · 09)	0 · 08 (0 · 04-0 · 17)	2 · 0	<0 · 001	<LOD^b^	0 · 05

Data are geometric means (25^th^-75^th^ percentile) of urinary concentrations in μg/L

^#^ *P*-values obtained by contrasting preeclamptic patients and controls in a one-way ANOVA on log-transformed values

^†^Upper reference limit in μg/L for the general US population according to NHANES (US National Health and Nutrition Examination Survey) as published by Paschal et al. [13] (^a^, P90) and Komaromy-Hiller et al. [14] (^b^, P87 · 5)

^‡^Upper reference limit in μg/L for the general Belgian population, data from Hoet et al. [15] (upper limit of 90 % confidence interval of P97 · 5)

When metal concentrations were expressed per gram creatinine, the values remained significantly higher in the preeclamptic group for all but four metals (Li, Mo, V, and Tl) (Additional file 1: Table S4). However, sample size was considerably smaller for the creatinine-corrected analysis (*N* = 59 and *N* = 63 for the rainy season and dry season, respectively), because many urine samples with too low creatinine (<0.3 g/L) had to be excluded.

Nevertheless, collection of total urine production in 24 h allowed us to calculate the daily amount of metals excreted in urine (μg/day). Diuresis itself differed markedly between the two study groups: on average, women with preeclampsia produced 275 mL (23 %) less urine in 24 h than control women (Table 4). Using the amount of metals excreted per day, the groups no longer differed for V, As, Se, Mo and Tl, in addition to Li, but the daily excretion of 13 elements remained significantly higher in preeclamptic women than in controls, by less than two-fold for Al, Te, and U, by 2- to 5-fold for Cr, Mn, Co, Ni, Cu, Cd, and Sb, and by 6- to 7-fold for Zn, Sn, and Pb (Table 4).

**Table 4:** Daily urinary metal excretion (in μg/day) in pregnant women with or without preeclampsia in Kinshasa

	Control (*N* = 88)	Preeclamptic (*N* = 88)	Fold diff.	*P* ^#^	Upper reference limit NHANES^a,b^
24 h diuresis (mL)	1,182 (925-1,415)	907 (695-1,100)		<0 · 001	N/A
Li	7 · 17 (3 · 68-13 · 3)	5 · 98 (3 · 76-8 · 56)	0 · 9	0 · 10	
Al	63 · 0 (21 · 5-220)	113 (46-352)	1 · 8	0 · 009	60 · 8
V	1 · 64 (1 · 00-2 · 50)	1 · 78 (1 · 02-2 · 99)	1 · 1	0 · 43	
Cr	1 · 00 (0 · 37-3 · 39)	3 · 89 (0 · 81-20 · 1)	3 · 9	<0 · 001	6 · 7
Mn	10 · 5 (2 · 38-29 · 0)	37 · 9 (4 · 4-274)	3 · 6	0 · 001	
Co	0 · 61 (0 · 24-1 · 73)	1 · 76 (0 · 67-4 · 01)	2 · 9	<0 · 001	
Ni	4 · 71 (2 · 47-10 · 2)	11 · 8 (5 · 29-20 · 8)	2 · 5	<0 · 001	10 · 7
Cu	39 · 2 (11 · 2-140 · 1)	193 (64-530)	4 · 9	<0 · 001	148 · 0
Zn	714 (194-1,074)	4,993 (916-32,094)	7 · 0	<0 · 001	1657 · 5
As	30 · 5 (15 · 0-59 · 3)	40 · 0 (23 · 3-78 · 7)	1 · 3	0 · 051	70 · 7
Se	31 · 0 (17 · 8-63 · 3)	38 · 0 (20 · 7-62 · 4)	1 · 2	0 · 15	
Mo	15 · 1 (8 · 0-33 · 8)	16 · 3 (7 · 4-31 · 1)	1 · 1	0 · 64	
Cd	0 · 61 (0 · 32-0 · 78)	1 · 51 (0 · 59-2 · 73)	2 · 5	<0 · 001	2 · 1
Sn	1 · 42 (0 · 51-3 · 05)	9 · 22 (1 · 78-45 · 6)	6 · 5	<0 · 001	
Sb	0 · 52 (0 · 14-1 · 66)	1 · 66 (0 · 62-5 · 69)	3 · 2	<0 · 001	
Te	0 · 12 (0 · 07-0 · 21)	0 · 19 (0 · 09-0 · 23)	1 · 6	0 · 018	
Ba	13 · 3 (5 · 9-28 · 9)	29 · 6 (6 · 6-93 · 7)	2 · 2	0 · 001	
Tl	0 · 26 (0 · 15-0 · 50)	0 · 30 (0 · 20-0 · 49)	1 · 2	0 · 22	<LOD
Pb	9 · 09 (3 · 15-20 · 5)	60 · 9 (8 · 3-345)	6 · 7	<0 · 001	8 · 0
U	0 · 05 (0 · 02-0 · 12)	0 · 07 (0 · 03-0 · 13)	1 · 5	0 · 023	

Data shown are geometric means (25^th^-75^th^ percentile) of 24 h excreted quantities in μg/day

^#^ *P*-values obtained by contrasting preeclamptic patients and controls in a one-way ANOVA on log-transformed values

^a^Upper reference limit in μg/day for the general US population according to NHANES, the US National Health and Nutrition Examination Survey, data from Komaromy-Hiller et al. [14] (P87 · 5)

^b^No data expressed in μg/day are available for Belgium

Within the group of preeclamptic patients, there were three women with eclampsia in each season. These six women did not differ from the other women with preeclampsia with regard to personal characteristics and urinary metal values (data not shown). Therefore, no further differentiation was made between results for preeclamptic and eclamptic patients.

**Fig. 2:**
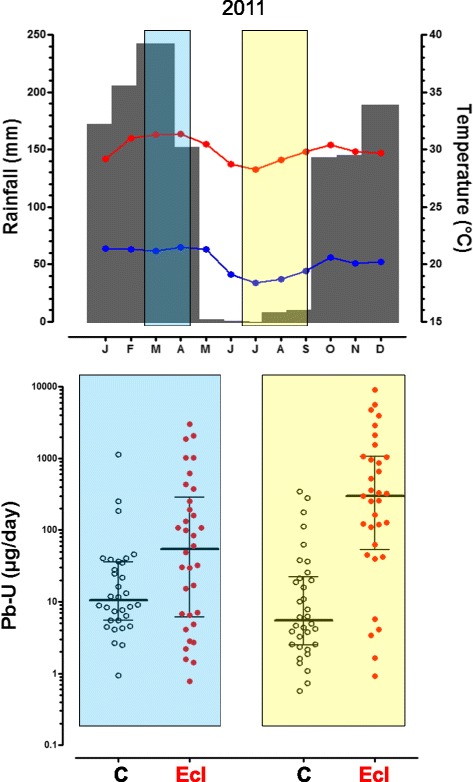
Meteorological data for 2011 in Kinshasa and daily urinary excretion of lead by pregnant women with and without preeclampsia. *Upper panel*: monthly rainfall (grey columns) and monthly averages of maximal (red line) and minimal (blue line) daily temperature in 2011 (Binza meteorological station). The light blue rectangle indicates the recruitment between March 1^st^ and April 18^th^ during the rainy season and the light yellow rectangle indicates the recruitment between July 1^st^ and September 2^nd^ during the dry season. *Lower panel*: individual values (with medians and 25^th^ and 75^th^ percentiles) of the daily urinary excretion of lead (Pb-U in μg/day) for pregnant women without (C, open symbols) and with preeclampsia (Ecl, full symbols), during the rainy season (light blue rectangle, left) and the dry season (light yellow rectangle, right). See Table 5 for significance levels

For 10 out of 20 elements, urinary concentrations (Additional file 1: Table S5) and daily excretions (Table 5) differed significantly between seasons, always being higher in the rainy season than in the dry season (regardless of the case or control status). Since the volume of urine was not affected by season, qualitatively similar seasonal effects were found for urinary concentrations and daily excretion. Preeclamptic women had significantly higher daily metal excretion than control women for 16 elements in the dry season, but for only five elements in the rainy season. Interactions between season and preeclampsia were significant for Li, As, Se, Sb, Te, Tl and Pb. Figure 2 illustrates that Pb excretion was 11.6-fold higher among preeclamptic women than control women in the dry season, but only 2.7-fold higher in the rainy season.

**Table 5:** Daily urinary metal excretion (in μg/day) according to season in pregnant women with or without preeclampsia in Kinshasa

	Rainy season (*N* = 67)		Dry season (*N* = 109)		*P* for group^#^	*P* for season^#^	*P* for interaction^#^
	Control (*N* = 33)	Preeclamptic (*N* =34)	Control (*N* =55)	Preeclamptic (*N* =54)			
Diuresis (mL)	1,225 (910-1,600)	916 (680-1,100)	1,156 (950-1,350)	901 (720-1,100)	<0 · 001	0 · 38	0 · 58
Li	14 · 3 (11 · 4-21 · 6)^c^	7 · 82 (4 · 25-12 · 9)^b^	4 · 75 (3 · 37-7 · 03)^a^	5 · 04 (3 · 47-7 · 27)^a^	0 · 005	<0 · 001	0 · 003
Al	144 (69 · 5-318)^b^	261 (71 · 0-907)^b^	38 · 4 (13 · 1-103 · 7)^a^	66 · 1 (23 · 0-178)^a^	0 · 005	<0 · 001	0 · 90
V	1 · 62 (0 · 98-2 · 89)^a^	1 · 66 (0 · 90-3 · 48)^a^	1 · 65 (1 · 13-2 · 48)^a^	1 · 86 (1 · 24-2 · 90)^a^	0 · 51	0 · 54	0 · 65
Cr	1 · 81 (0 · 63-4 · 38)^a,b^	4 · 36 (1 · 23-23 · 8)^b^	0 · 70 (0 · 19-1 · 93)^a^	3 · 62 (0 · 70-19 · 9)^b^	<0 · 001	0 · 038	0 · 16
Mn	17 · 5 (4 · 3-28 · 1)^a,b^	26 · 7 (4 · 13-110)^a,b^	7 · 74 (1 · 10-29 · 9)^a^	47 · 3 (7 · 63 -527)^b^	0 · 007	0 · 76	0 · 09
Co	1 · 10 (0 · 61-1 · 90)^b^	2 · 30 (0 · 69-4 · 53)^b^	0 · 43 (0 · 15-1 · 21)^a^	1 · 49 (0 · 63-3 · 78)^b^	<0 · 001	<0.001	0 · 21
Ni	6 · 55 (3 · 65-15 · 1)^a,b^	19 · 4 (6 · 26-90 · 7)^c^	3 · 87 (1 · 97-7 · 44)^a^	8 · 59 (4 · 71-13 · 0)^b^	<0 · 001	<0 · 001	0 · 42
Cu	45 · 9 (16 · 1-140)^a^	164 (81 · 2-425)^b^	35 · 7 (6 · 5-186)^a^	213 (53.9-757)^b^	<0 · 001	0 · 99	0 · 33
Zn	807 (362-1,053)^a^	5,220 (1,391-10,898)^b^	663 (143-1,107)^a^	4,856 (535-50,760)^b^	<0 · 001	0 · 71	0 · 86
As	59 · 9 (39 · 5-91 · 8)^c^	49 · 5 (25 · 3-86 · 0)^b,c^	20 · 4 (13 · 1-26 · 7)^a^	35 · 0 (20 · 5-60 · 5)^b^	0 · 18	<0 · 001	0 · 005
Se	55 · 4 (42 · 4-86 · 7)^b^	41 · 6 (30 · 3-77 · 3)^b^	21 · 9 (12 · 6-31 · 0)^a^	35 · 9 (19 · 3-59 · 7)^b^	0 · 14	<0 · 001	0 · 005
Mo	24 · 3 (14 · 2-50 · 7)^b^	17 · 4 (10 · 0-37 · 0)^a,b^	11 · 4 (6 · 10-14 · 4)^a^	15 · 7 (7 · 1-25 · 8)^a,b^	0 · 97	0 · 010	0 · 051
Cd	0 · 73 (0 · 47-0 · 98)^a^	1 · 62 (0 · 61-3 · 27)^b^	0 · 54 (0 · 29-0 · 55)^a^	1 · 45 (0 · 59-2 · 64)^b^	<0 · 001	0 · 30	0 · 63
Sn	1 · 77 (0 · 78-6 · 26)^a^	12 · 2 (4 · 6-60 · 2)^b^	1 · 25 (0 · 44-2 · 28)^a^	7 · 74 (1 · 35-24 · 0)^b^	<0 · 001	0 · 20	0 · 87
Sb	2 · 15 (1 · 34-3 · 95)^c^	3 · 22 (1 · 28-7 · 80)^c^	0 · 22 (0 · 13-0 · 40)^a^	1 · 10 (0 · 35-3 · 23)^b^	<0 · 001	<0 · 001	0 · 004
Te	0 · 15 (0 · 13-0 · 21)^a,b^	0 · 11 (0 · 09-0 · 18)^a^	0 · 11 (0 · 06-0 · 23)^a^	0 · 27 (0 · 10-0 · 38)^b^	0 · 10	0 · 11	0 · 002
Ba	14 · 0 (6 · 3-26 · 0)^a,b^	35 · 5 (7 · 46-104)^b^	13 · 0 (4 · 7-30 · 7)^a^	26 · 5 (5 · 4-91 · 2)^b^	0 · 001	0 · 44	0 · 66
Tl	0 · 42 (0 · 24-0 · 73)^b^	0 · 33 (0 · 20-0 · 62)^b^	0 · 20 (0 · 13-0 · 35)^a^	0 · 29 (0 · 23-0 · 46)^b^	0 · 55	<0 · 001	0 · 010
Pb	17 · 0 (5 · 5-36 · 1)^a,b^	46 · 3 (6 · 5-254)^b^	6 · 24 (2 · 52-9 · 94)^a^	72 · 4 (9 · 3-358)^b^	<0 · 001	0 · 37	0 · 019
U	0 · 12 (0 · 08-0 · 27)^c^	0 · 14 (0 · 08-0 · 34)^c^	0 · 03 (0 · 02-0 · 05)^a^	0 · 05 (0 · 02-0 · 11)^b^	0 · 028	<0 · 001	0 · 13

Data are geometric means (25^th^–75^th^ percentile) of daily urinary excretion in μg/day

^#^ *P*-values obtained by two-way ANOVA on log-transformed values ^a,b,c^ Values with the same letter in superscript do not differ significantly from each other, according to Tukey’s post-hoc test

The daily excreted quantities of metals were highly correlated among each other, except for Li and Mo (Additional file 1: Table S6). The subsequent PCA revealed that the eigenvalue of the first PC accounted for 43 % of the total variance, and three eigenvalues explained 67 % of the total variance. The first PC was positively correlated with all 20 metals (all *p* < 0.001), indicating that this PC can be considered as a composite metal excretion variable. A two-way ANOVA, similar to those performed for the individual elements, resulted in a highly significant group effect (*p* < 0.001) with higher values for preeclamptics than for control subjects, a highly significant season effect (*p* < 0.001) with higher values during the rainy season, and a significant interaction effect (*p* = 0.038), revealing that the higher values in patients occurred in the dry season and not in the rainy season.

## Discussion

We demonstrated a very high exposure to pollutant metals in pregnant women in Kinshasa, and observed that women with preeclampsia excreted higher quantities of metals than pregnant women without preeclampsia. Moreover, the differences in metal excretion between the two groups were less pronounced in the rainy season than in the dry season, when the incidence of preeclampsia is highest.

We studied a relatively large group of women hospitalized for preeclampsia, who were compared with an equal number of well-matched healthy pregnant women without preeclampsia. Novel features of our study include the use of ICP-MS (allowing the measurement of several elements in one sample), the collection of 24-h urine (thus avoiding the need for correcting for urine dilution), and the enrolment over two different seasons in order to understand the seasonality of preeclampsia.

An obvious limitation of our study is its case-control design and the absence of measurements before (or after) the third trimester of pregnancy, which precludes drawing causal inferences. Other limitations are the absence of blood measurements, and the lack of dietary or environmental exposure data.

One could criticize that our control group did not necessarily reflect the source population of our cases, since it consisted of healthy pregnant women recruited from an antenatal clinic, rather than pregnant women hospitalized for reasons other than preeclampsia (e.g. malaria, diabetes, or hypertension without proteinuria). However, our control subjects came from the same geographical areas as the cases, and they were closely matched for important factors such as age, parity and gestational age. So, our control group may be considered a suitable group of community controls from the city of Kinshasa. From a methodological point, a relative limitation is that we did not perform a matched analysis of the data, even though our controls had been selected by individual matching with cases. Whilst this may have led to a bias towards the null for some metals, it does not affect our overall conclusions.

Another limitation is that our study was conducted in non-nulliparous women. A first pregnancy is a major risk factor for the occurrence of preeclampsia [3] and we wanted to avoid this dominant risk factor in order to increase the chances of detecting associations with other, possibly less influential environmental factors. The exclusion of nulliparous women limits the generalizability of our findings and further studies should be conducted to verify whether our observations also apply to primigravidae.

The average concentrations of metals or metalloids found in the urine of our control subjects were substantially higher than the upper limits published for adults from the general population in economically developed countries [13–15]. The high values found here corroborate findings previously reported for the general population of Kinshasa [6].

One caveat is that pregnancy itself may affect the urinary excretion of metals. Thus, Pb is well-known to be mobilized from its skeletal stores during pregnancy [16]. After an initial decrease during early pregnancy, blood Pb levels increase moderately during the third trimester of pregnancy, especially in older women and if calcium intake levels are low [17, 18]. A daily excretion of 0.8 to 5.9 μg Pb (geometric mean of 1.9 μg; compared with 9.1 μg in our control group) was found during pregnancy and post-partum in 13 Australian women [19]. Pregnant women from Lagos, Nigeria, had two to three-fold higher concentrations of Pb in blood and urine than nonpregnant women [20].

It is conceivable that pregnancy also affects the toxicokinetics of other metals besides Pb. However, although the reproductive and developmental toxicity of metals has received considerable attention [21], we found no human studies that evaluated pregnancy-related changes in the excretion of metals other than Pb [22].

The urinary metal concentrations were very high in our control subjects, but they were even higher in women with preeclampsia. A well-known issue when metals or other agents are measured in urine is how to correct for dilution. Usually, this is achieved by relating the concentrations of the analyte to that of creatinine, because it is assumed that the daily excretion of creatinine is constant and reasonably equal for all (normal) subjects [22, 23]. However, the validity of creatinine corrections has not been established during pregnancy, let alone in preeclampsia. Moreover, creatinine may be degraded when samples are not kept cold, as probably happened with one of the batches of the present study. We, therefore, chose not to rely on creatinine-corrected values, even though the differences in metal concentrations between the two groups also held when creatinine corrections were applied (see Additional file 1: Table S4).

However, since urine had been collected over 24 h, we were able to express our data in terms of daily excretion, which is probably the best option. As expected [24, 25], the women with preeclampsia produced substantially less urine than control women, thus reducing the differences between the two groups without, however, abolishing them. In the (hospitalized) women with preeclampsia, urine was collected via urinary catheter, whilst the control (outpatient) women collected their 24-h urine at home; the latter entails a risk of external contamination and of incomplete urine collections among controls, but both factors would tend to reduce (rather than spuriously create) the differences found in metal excretions between the two groups. Hence, the differences found between cases and controls are unlikely to be artifactual. Nevertheless, some of the increased metal concentrations in preeclamptic women may be due to proteinuria, since many metals are bound to serum proteins. We did not attempt to evaluate this with our material.

The hypothesis that preeclampsia may be linked with Pb exposure already dates from the nineteenth century and it was verified by showing ecological associations between Pb in drinking water and the incidence of eclampsia in Britain in the early twentieth century [26]. In the USA, the risk of pregnancy-induced hypertension was shown to be associated with airborne lead concentrations averaged at state level [27]. A recent systematic review found a significant association between blood Pb and gestational hypertension or preeclampsia in six out of nine identified epidemiological studies [5]. Studies from Iran [28], Nigeria [29], and Egypt [30] also described higher blood Pb in preeclampsia. The simplest explanation for these findings is that the higher levels of Pb in blood (or in urine, as in our study) in women with preeclampsia, reflect a higher past or ongoing exposure to Pb. Nevertheless, an alternative interpretation is that preeclampsia is accompanied by a higher release of Pb from body stores (or a higher leak through injured kidneys), than in normal pregnancy. Such possible “reverse causation” does not appear to have been investigated. In other words, like other cross-sectional studies, our study cannot tell whether (or to what extent) the much higher urinary (or indeed blood) Pb levels found in preeclamptic women indicate that Pb is involved in the pathogenesis of the disease or whether preeclampsia simply leads to higher levels of Pb in blood and urine. To solve the issue of causality would require assessing the incidence of preeclampsia in a longitudinal design. Observations made in the wake of the Hurricanes Katrina and Rita in New Orleans, argue against reverse causation because parallel changes occurred (at neighbourhood level) between soil concentrations of Pb and the incidence of eclampsia [31].

It is generally stated that blood Pb reflects body burden better than urine Pb [22]. However, this is valid in steady-state conditions only and probably not when Pb is being mobilized from bone, as in pregnancy. Thus, whilst measurements of blood Pb would have been desirable, 24-h excretion values are probably more relevant to assess differences in internal exposure to Pb between preeclampsia and normal pregnancy, whether this difference is due to a higher extraction of Pb from body stores, to a higher past or ongoing exposure to Pb, or a combination of both.

Why Pb may be instrumental in causing preeclampsia has not been elucidated. Most authors have evoked the known nephrotoxicity and vascular endothelial toxicity of Pb [5]. We did not evaluate whether the excretion of Pb (or any other element) correlated quantitatively with indices of severity (such as proteinuria or arterial pressure), but we did not find higher urinary metal values in the six women with full-blown eclampsia compared to those with preeclampsia only.

Other metals have not received as much attention as Pb in the context of preeclampsia. Because Pb and the other trace elements were highly correlated in urine, most metals were also increased among preeclamptic women. This could imply that “metal exposure” (as captured by our principal component analysis) is related to preeclampsia, with Pb being essentially a marker of risk rather than a causal agent. However, of all the measured toxic elements, Pb exhibited the strongest contrast between preeclampsia and no preeclampsia, although Sn and Zn also differed as much between the two groups.

Like Pb, Sn is stored in bone but inorganic Sn compounds are not well absorbed and much less toxic than Pb [32] and we found no studies investigating the relation between Sn and preeclampsia.

In contrast, various studies have investigated the possible implication of Zn, Se, Cu, or Mn in preeclampsia because these trace metals are essential micronutrients involved in anti-oxidant defence [33–35]. Consequently, *deficiencies* in these essential elements, rather than excesses, are of concern. Accordingly, serum concentrations of Zn have been found to be decreased in preeclampsia [34]. Similarly, a low Se status has been implicated in reproductive and obstetric complications, including preeclampsia [33, 34, 36]. In contrast, serum Cu has been found to be decreased [37–40], unchanged [41] or increased [42–45] in preeclampsia. One study reported a decrease in Mn in umbilical blood from neonates born to mothers with preeclampsia [46]. In our study, preeclamptic women had markedly elevated urinary values for Zn, Cu and Mn, when compared with either the control group or upper reference limits. For Se, the differences between the two groups were less marked and the values were not particularly high compared to reference values [14]. In the absence of blood or serum concentrations of these essential metals, we cannot directly compare our findings with those of the literature.

Cd excretion was 2.5 fold higher in preeclamptic women than in control women, possibly due to proteinuria, but we found almost no evidence for a role of Cd in the pathogenesis of preeclampsia [28]. The urinary values of As were not very high and the differences between the groups were not pronounced. Although As is vasculotoxic [47], As has not been associated with preeclampsia.

The incidence of preeclampsia in Kinshasa is twice as high in the dry season than in the rainy season [2]. A possible explanation is the lower availability and, hence, reduced consumption of fresh vegetables and fruit during the dry season, especially among disadvantaged people [4, 48]. However, another feature of the dry season in countries with few hardened surfaces is a much higher aerial dust suspension than in the rainy season, thus leading to more pollutant exposure via the air and through contamination of food, clothes and indoor surfaces. Seasonal influences mediated by soil exposure have been well documented for Pb, especially among young children in the USA [7, 49–52]. In pregnant women from Mexico City, blood Pb was higher during fall and winter (dry and cold) and lower during spring and summer (rainy) [53]. No seasonal effects were apparent for blood Pb in Australian children and women [54].

We observed significant seasonal differences for the urinary excretion of various metals but, against expectation, the daily excretion of metals was not highest during the dry season. Thus, in the control group, Pb tended to be higher in the rainy season (14.4 μg/L and 17.0 μg/day) than in the dry season (5.6 μg/L and 6.2 μg/day). We have no explanations for this counter-intuitive observation. We speculate that during pregnancy the urinary excretion of Pb (and other metals) is dominated by the amount that is being extracted from the skeletal stores and, hence, by the exposure of the *previous* months, rather than by ongoing exposure. Among women with preeclampsia, the urinary values were also not higher in the dry season than in the rainy season, but the seasonal differences tended to be less pronounced than in normal women. This led to significant or nearly significant interactions between group and season for several elements. For Pb, the relative difference between cases and controls was much larger in the dry season than in the rainy season. Admittedly, the interpretation of these interactions with season is not straightforward, but the observed seasonal differences do suggest that metal exposure differs between seasons in Kinshasa. Whether and how this relates to the causal pathway with preeclampsia remains to be established by prospective studies, which should include assessments of environmental and dietary sources of exposure.

Indeed, we do not know the exact sources of toxic metals in our population. African geophagic soil samples may contain high levels of lead [55], but in our study the proportions of women reporting geophagy did not differ between preeclamptic women (32 %) and control women (27 %). High blood Pb levels in adults and children from urban Kinshasa have been attributed to leaded gasoline and informal car battery recycling in some residences [56]. We have some indications that the women with preeclampsia had more opportunities for exposure through artisanal activities, such as battery recycling, car painting or metal working close to their homes. The devastating effects of artisanal activities, such as recycling batteries or processing gold ore, for children in African communities have been demonstrated in Senegal [57] and Nigeria [58].

## Conclusions

Our study provides novel evidence for an excessive exposure to trace metals among pregnant women in a large African city, Kinshasa. The markedly increased urinary excretion of metals, especially Pb, observed in preeclampsia may be related to the high incidence of preeclampsia in Kinshasa. An important public health issue is to ascertain the main sources of exposure to toxic metals in Kinshasa and to take preventive measures to avoid further contamination of pregnant women and children.

## Additional file

Additional file 1: Toxic metals and preeclampsia in Kinshasa: additional data. (DOCX 85 kb)

## Abbreviations

ANOVA: analysis of variance
BMI: body mass index
CI: confidence interval
GM: geometric mean
ICP-MS: inductively coupled plasma mass spectrometry
LOD: limit of detection
NHANES: national health and nutrition examination survey
OR: odds ratio
PC: principal component
PCA: principal component analysis
