# Supplementary material for: Preeclampsia and toxic metals: a case-control study in Kinshasa, DR Congo
Source: Environ Health. 2016 Apr 5;15:48. doi: 10.1186/s12940-016-0132-1 (PMC4820935; doi:10.1186/s12940-016-0132-1)
Supplement: Additional file 1: — Toxic metals and preeclampsia in Kinshasa: additional data. (DOCX 85 kb) [file 12940_2016_132_MOESM1_ESM.docx]

**High and season-dependent urinary excretion of toxic metals in women with preeclampsia. A case-control study in Kinshasa, DR Congo**

Jean-Pierre Elongi Moyene, Hans Scheers, Barthélémy Tandu-Umba, Vincent Haufroid, Baudouin Buassa-bu-Tsumbu, Fons Verdonck, Bernard Spitz, Benoit Nemery

**Analytical methods**

The urine samples were analyzed at the Louvain centre for Toxicology and Applied Pharmacology (Université catholique de Louvain, Belgium) without knowledge of their provenance (case or control). In all urine samples, the concentration of 24 metals or metalloids (all called “metals” hereafter) were quantified by means of ICP-MS with an Agilent 7500 ce instrument, as described in previous publications [1,2]. Urine specimens (500 µl) were diluted quantitatively (1+9) with a HNO_3_ 1%, HCl 0·5% solution containing Sc, Ge, Rh and Ir as internal standards. Li, Be, Al, Mo, Cd, In, Sn, Sb, Te, Ba, Pt, Tl, Pb, Bi and U were analyzed using no-gas mode, and V, Cr, Mn, Co, Ni, Cu, Zn, As and Se were analyzed using helium mode. (Mercury was not measured).

Using this validated method, the laboratory has obtained successful results in external quality assessment schemes organized by the Institute for Occupational, Environmental and Social Medicine of the University of Erlangen, Germany (G-EQUAS program), and by the Institut National de Santé Publique, Québec (PCI and QMEQAS programs). Moreover, the laboratory possesses an ISO15189 certification for the measurements of 20 metals in urine (Be, Al, V, Cr, Mn, Co, Ni, Cu, Zn, As, Se, Mo, Cd, Sn, Sb, Te, Ba, Tl, Pb and Bi).

**Supplemental tables S1-S6**

**Table S1.** **Details on the distribution of urinary metal concentrations (in µg/L) in pregnant women in Kinshasa (control group, no preeclampsia; N=88).**

|  | *LOD* | % below LOD | Min | P25 | P50 (median) | P75 | P95 | Max |
| --- | --- | --- | --- | --- | --- | --- | --- | --- |
| Li | *0·044* | 0·0 | 1·15 | 3·34 | 6·31 | 11·8 | 20·2 | 35·3 |
| Be | *0·015* | 98·9 | <LOD | <LOD | <LOD | <LOD | <LOD | 0·017 |
| Al | *1·572* | 0·0 | 2·78 | 21·9 | 64·2 | 172 | 361 | 902 |
| V | *0·120* | 0·0 | 0·48 | 0·97 | 1·36 | 1·90 | 4·10 | 10·7 |
| Cr | *0·061* | 3·4 | <LOD | 0·39 | 0·70 | 2·46 | 8·54 | 38·8 |
| Mn | *0·046* | 2·3 | <LOD | 2·06 | 6·97 | 27·7 | 1,107 | 2,474 |
| Co | *0·018* | 0·0 | 0·030 | 0·21 | 0·63 | 1·37 | 3·16 | 4·58 |
| Ni | *0·277* | 3·4 | <LOD | 2·42 | 4·23 | 8·98 | 16·5 | 64·1 |
| Cu | *0·142* | 0·0 | 1·31 | 10·6 | 22·7 | 133 | 502 | 6,508 |
| Zn | *0·596* | 0·0 | 10·6 | 186 | 417 | 1129 | 76,440 | 710,200 |
| As | *0·073* | 0·0 | 4·83 | 13·4 | 24·8 | 51·6 | 111 | 209 |
| Se | *0·712* | 0·0 | 4·31 | 13·5 | 29·0 | 54·3 | 81·1 | 207 |
| Mo | *0·051* | 0·0 | 0·66 | 7·19 | 10·2 | 29·8 | 113 | 186 |
| Cd | *0·015* | 0·0 | 0·079 | 0·29 | 0·43 | 0·68 | 12·5 | 77·2 |
| In | *0·020* | 100 | <LOD | <LOD | <LOD | <LOD | <LOD | <LOD |
| Sn | *0·032* | 2·3 | <LOD | 0·43 | 1·01 | 2·60 | 56·6 | 543 |
| Sb | *0·015* | 1·1 | <LOD | 0·14 | 0·36 | 1·57 | 4·53 | 43·5 |
| Te | *0·030* | 10·2 | <LOD | 0·066 | 0·12 | 0·19 | 0·34 | 7·30 |
| Ba | *0·138* | 0·0 | 0·75 | 5·52 | 10·4 | 24·9 | 143 | 570 |
| Pt | *0·061* | 96·6 | <LOD | <LOD | <LOD | <LOD | <LOD | 0·10 |
| Tl | *0·074* | 9·1 | <LOD | 0·15 | 0·21 | 0·45 | 0·71 | 0·89 |
| Pb | *0·032* | 0·0 | 0·56 | 3·14 | 6·03 | 13·8 | 205 | 2,613 |
| Bi | *0·018* | 75·0 | <LOD | <LOD | <LOD | <LOD | 0·044 | 0·11 |
| U | *0·008* | 4·6 | <LOD | 0·018 | 0·039 | 0·093 | 0·29 | 0·50 |

*LOD* is Limit of Detection· Min is lowest value; P25, P50, P75, P95 are percentiles; Max is highest value·

**Table S2.** **Details on the distribution of urinary metal excretion (in µg/day) in pregnant women in Kinshasa (control group, no preeclampsia; N=88)**

|  | Min | P25 | P50 (median) | P75 | P95 | Max |
| --- | --- | --- | --- | --- | --- | --- |
| Li | 1·02 | 3·68 | 6·97 | 13·3 | 29·9 | 38·4 |
| Al | 3·95 | 21·5 | 67·3 | 220 | 480 | 902 |
| V | 0·40 | 1·00 | 1·46 | 2·50 | 5·81 | 13·9 |
| Cr | 0·034 | 0·37 | 0·81 | 3·39 | 11·1 | 49·3 |
| Mn | 0·025 | 2·38 | 8·53 | 29·0 | 1,771 | 3,538 |
| Co | 0·027 | 0·24 | 0·68 | 1·73 | 3·74 | 5·76 |
| Ni | 0·11 | 2·47 | 4·49 | 10·2 | 27·5 | 78·9 |
| Cu | 1·87 | 11·2 | 29·6 | 140 | 844 | 9,111 |
| Zn | 15·2 | 194 | 459 | 1,074 | 91,104 | 858,900 |
| As | 3·93 | 15·0 | 26·8 | 59·3 | 145 | 217 |
| Se | 3·83 | 17·8 | 30·6 | 63·3 | 104 | 269 |
| Mo | 0·51 | 7·99 | 12·7 | 33·9 | 126 | 184 |
| Cd | 0·095 | 0·32 | 0·47 | 0·78 | 13·5 | 100 |
| Sn | 0·015 | 0·51 | 1·26 | 3·05 | 95·6 | 565 |
| Sb | 0·009 | 0·14 | 0·43 | 1·66 | 5·56 | 69·6 |
| Te | 0·013 | 0·074 | 0·13 | 0·21 | 0·37 | 6·57 |
| Ba | 0·83 | 5·90 | 12·7 | 28·9 | 157 | 593 |
| Tl | 0·033 | 0·15 | 0·25 | 0·50 | 1·04 | 1·44 |
| Pb | 0·56 | 3·15 | 6·20 | 20·5 | 253 | 4,180 |
| U | 0·003 | 0·020 | 0·046 | 0·12 | 0·35 | 0·50 |

Min is lowest value (taking ½ LOD if < LOD); P25, P50, P75, P95 are percentiles; Max is highest value.

**Table S3.** **Details on the distribution of urinary metal concentrations (in µg/g creatinine) in pregnant women in Kinshasa (control group, no preeclampsia, N=64*).**

|  | Min· | P25 | P50 (median) | P75 | P95 | Max· |
| --- | --- | --- | --- | --- | --- | --- |
| Li | 1·47 | 4·86 | 6·20 | 10·4 | 18·4 | 37·5 |
| Al | 4·17 | 23·9 | 59·1 | 123 | 398 | 594 |
| V | 0·32 | 0·62 | 1·02 | 2·24 | 4·64 | 7·14 |
| Cr | 0·029 | 0·28 | 0·52 | 2·37 | 7·18 | 42·2 |
| Mn | 0·019 | 1·40 | 4·25 | 11·5 | 179 | 1,460 |
| Co | 0·056 | 0·27 | 0·50 | 1·04 | 1·92 | 2·89 |
| Ni | 0·19 | 2·34 | 3·64 | 6·97 | 17·6 | 20·7 |
| Cu | 1·30 | 6·79 | 14·3 | 69·7 | 324 | 1014 |
| Zn | 49·6 | 171 | 394 | 791 | 166,811 | 776,667 |
| As | 6·95 | 20·7 | 32·2 | 48·6 | 80·2 | 119 |
| Se | 16·1 | 25·0 | 31·8 | 42·0 | 54·3 | 193 |
| Mo | 3·00 | 8·45 | 15·5 | 24·9 | 67·8 | 94·9 |
| Cd | 0·15 | 0·26 | 0·33 | 0·71 | 15·3 | 72·1 |
| Sn | 0·023 | 0·33 | 0·99 | 2·67 | 120 | 315 |
| Sb | 0·023 | 0·14 | 0·41 | 1·46 | 3·95 | 65·0 |
| Te | 0·019 | 0·069 | 0·098 | 0·16 | 0·36 | 3·63 |
| Ba | 1·07 | 4·52 | 10·9 | 21·4 | 247 | 438 |
| Tl | 0·077 | 0·19 | 0·26 | 0·33 | 0·57 | 1·02 |
| Pb | 1·07 | 2·45 | 4·03 | 9·22 | 61·3 | 3,900 |
| U | 0·002 | 0·018 | 0·037 | 0·090 | 0·21 | 0·62 |

*Subjects with urinary creatinine below 0·3 g/L and above 3 g/L have been excluded.

Min is lowest value (taking ½ LOD if < LOD); P25, P50, P75, P95 are percentiles; Max is highest value.

**Table S4.** **Urinary metal concentrations (in µg/g creatinine) in pregnant women with or without preeclampsia in Kinshasa.**

|  | Controls (N=64) | Preeclamptics  (N=58) | Fold diff· | P^#^ | Upper ref· limit 1 ^†^ | Upper ref· limit 2 ^‡^ |
| --- | --- | --- | --- | --- | --- | --- |
| Creatinine· (g/L) | 1·34 (0·74-1·90) | 1·44 (0·91-1·97) |  | 0·73 |  |  |
|  |  |  |  |  |  |  |
| Li | 7·11 (4·86-10·4) | 6·39 (4·61-9·19) | 0·9 | 0·34 |  | 100 |
| Al | 56·4 (23·9-123) | 130 (43·7-490) | 2·3 | 0·001 | 60·8^b^ | 10 |
| V | 1·18 (0·62-2·24) | 1·57 (0·77-3·24) | 1·3 | 0·09 |  | 2 |
| Cr | 0·72 (0·28-2·37) | 4·33 (0·88-20·3) | 6·0 | <0·001 | 0·60^a^, 4·5 ^b^ | 0·35 |
| Mn | 5·08 (1·40-11·5) | 28·0 (3·73-157) | 5·5 | <0·001 | 2·42^a^ | 1 |
| Co | 0·48 (0·27-1·04) | 2·08 (0·85-5·31) | 4·3 | <0·001 | 4·72^a^, 2·0 ^b^ | 1·3 |
| Ni | 4·10 (2·34-6·97) | 13·8 (5·36-33·3) | 3·4 | <0·001 | 14·2 ^b^ | 5 |
| Cu | 23·4 (6·79-69·7) | 143 (37.6-425) | 6·1 | <0·001 | 87·2 ^b^ | 14 |
| Zn | 647 (172-791) | 5,873 (1,046-31,034) | 9·1 | <0·001 | 938·4 ^b^ | 770 |
| As | 31·1 (20·7-48·6) | 48·4 (23·1-89·4) | 1·6 | 0·002 | 62·0 ^b^ | 260 |
| Se | 32·7 (25·0-50·0) | 47·1 (27·6-60·2) | 1·4 | 0·002 | 131·5 ^b^ | 40 |
| Mo | 15·0 (8·45-24·9) | 18·6 (9·76-30·1) | 1·2 | 0·19 | 133·8^a^, 69·0 ^b^ | 100 |
| Cd | 0·54 (0·26-0·71) | 1·80 (0·67-4·31) | 3·3 | <0·001 | 1·4 ^b^ | 1 |
| Sn | 1·26 (0·33-2·67) | 11·5 (2·03-70·8) | 9·1 | <0·001 | 39·9 ^b^ | 2·5 |
| Sb | 0·50 (0·14-1·46) | 2·14 (0·87-6·44) | 4·2 | <0·001 | 2·82^a^, <LOD ^b^ | 0·25 |
| Te | 0·11 (0·069-0·16) | 0·25 (0·10-0·43) | 2·2 | <0·001 | <LOD ^b^ | 0·5 |
| Ba | 11·2 (4·52-21·4) | 44·4 (14·0-179) | 4·0 | <0·001 | 7·52^a^, 9·3 ^b^ | 8 |
| Tl | 0·26 (0·19-0·33) | 0·31 (0·19-0·44) | 1·2 | 0·15 | 0·76^a^, <LOD ^b^ | 0·5 |
| Pb | 6·05 (2·45-9·22) | 36·9 (5·61-167) | 6·1 | <0·001 | 5·50^a^, 6·1 ^b^ | 3 |
| U | 0·041 (0·018-0·090) | 0·078 (0·031-0·16) | 1·9 | 0·003 | <LOD ^b^ | 0·04 |

Data are geometric means (25^th^-75^th^ percentile) of urinary concentrations in µg/g creatinine (only subjects with creatinine concentrations between 0·3 g/L and 3·0 g/L.

^#^ P-values obtained by contrasting preeclamptic patients and controls in a one-way ANOVA and adjusting with the Benjamini-Hochberg method for multiple testing (20 ANOVA tests on 20 metals).

^†^ Upper reference limit in µg/g creatinine for the general U·S· population according to NHANES (US National Health and Nutrition Examination Survey) as published by Paschal et al. [3] (^a^, P90) and Komaromy-Hiller et al. [4] (^b^, P87·5) [3,4]

^‡^ Upper reference limit in µg/g creatinine for the general Belgian population, data from Hoet et al.[1] (upper limit of 90% confidence interval of P97·5).

**Table S5.** **Urinary metal concentrations (in µg/L) according to season in pregnant women with or without preeclampsia in Kinshasa**

|  | Rainy season (N=67) | | Dry season (N=109) | | P for group^#^ | P for season ^#^ | P for inter­action^#^ |
| --- | --- | --- | --- | --- | --- | --- | --- |
|  | Control  (N=33) | Preeclamptic  (N =34) | Control  (N =55) | Preeclamptic  (N =54) |  |  |  |
| Li | 12·1 (11·2-15·8)^c^ | 9·19 (7·12-14·7)^c^ | 4·26 (3·12-6·34)^a^ | 5·92 (4·47-8·14)^b^ | 0·76 | <0·001 | 0·018 |
| Al | 122 (56·4-267)^b^ | 307 (94·4-772)^c^ | 34·4 (10·9-106)^a^ | 77·6 (30·8-202)^b^ | <0·001 | <0·001 | 0·89 |
| V | 1·37 (0·84-1·93)^a^ | 1·95 (1·05-3·34)^a,b^ | 1·48 (1·06-1·87)^a^ | 2·19 (1·48-3·63)^b^ | <0·001 | 0·49 | 0·89 |
| Cr | 1·53 (0·60-3·25)^a^ | 5·12 (1·62-24·2)^b^ | 0·63 (0·20-1·91)^a^ | 4·25 (0·80-24·4)^b^ | <0·001 | 0·08 | 0·35 |
| Mn | 14·8 (3·74-22·1) ^a,b^ | 31·4 (5·27-128)^b^ | 6·95 (1·40-33·3)^a^ | 55·5 (8·34-675)^b^ | <0·001 | 0·86 | 0·22 |
| Co | 0·93 (0·53-1·56)^b^ | 2·70 (0·72-4·98)^c^ | 0·39 (0·12-1·08)^a^ | 1·75 (0·76-3·93)^b,c^ | <0·001 | 0·002 | 0·42 |
| Ni | 5·55 (3·56-11·9)^a,b^ | 22·8 (7·90-85·9)^c^ | 3·47 (1·77-6·48)^a^ | 10·1 (5·96-19·1)^b^ | <0·001 | <0·001 | 0·48 |
| Cu | 38·9 (15·1-99·6)^a^ | 193(93·0-390)^b^ | 32·1 (6·10-148)^a^ | 250 (62·2-958)^b^ | <0·001 | 0·90 | 0·56 |
| Zn | 683 (365-744)^a^ | 6,131 (1,469-14,380)^b^ | 596 (118-1,181)^a^ | 5,700 (487-61,010)^b^ | <0·001 | 0·86 | 0·93 |
| As | 50·8(37·0-73·4)^b^ | 58·1 (36·1-81·9)^b^ | 18·3 (11·7-25·2)^a^ | 41·0 (22·0-74·1)^b^ | <0·001 | <0·001 | 0·034 |
| Se | 46·9 (39·7-71·3)^b^ | 48·8 (32·4-68·7)^b^ | 19·6 (11·9-31·0)^a^ | 42·1 (20·8-72·1)^b^ | 0·003 | <0·001 | 0·034 |
| Mo | 20·6 (11·7-44·2)^b^ | 20·5 (15·0-40·5)^b^ | 10·2 (5·63-12·6)^a^ | 18·4 (8·51-33·3)^b^ | 0·078 | 0·027 | 0·17 |
| Cd | 0·62 (0·41-0·71)^a^ | 1·90 (0·73-3·90)^b^ | 0·49 (0·24-0·63)^a^ | 1·70 (0·71-2·69)^b^ | <0·001 | 0·51 | 0·89 |
| Sn | 1·50 (0·66-5·20)^a^ | 14·3 (4·25-66·3)^b^ | 1·12 (0·36-2·21)^a^ | 9·09 (1·76-30·1)^b^ | <0·001 | 0·37 | 0·89 |
| Sb | 1·82 (1·00-3·15)^b,c^ | 3·78 (1·39-9·19)^c^ | 0·20 (0·11-0·35)^a^ | 1·29 (0·44-4·04)^b^ | <0·001 | <0·001 | 0·034 |
| Te | 0·12 (0·097-0·19)^a^ | 0·13 (0·11-0·20)^a^ | 0·10 (0·054-0·19)^a^ | 0·32 (0·13-0·39)^b^ | 0·002 | 0·13 | 0·034 |
| Ba | 11·9 (5·97-20·0)^a^ | 41·7 (12·5-136)^b^ | 11·6 (4·56-26·0)^a^ | 31·1 (7·03-105)^b^ | <0·001 | 0·60 | 0·76 |
| Tl | 0·35 (0·21-0·55)^b^ | 0·38 (0·23-0·61)^b^ | 0·18 (0·14-0·38)^a^ | 0·34 (0·27-0·56)^b^ | 0·002 | 0·002 | 0·055 |
| Pb | 14·4 (5·25-24·0)^a^ | 54·4 (5·23-246)^b^ | 5·60 (2·61-12·1)^a^ | 85·0 (9·67-700)^b^ | <0·001 | 0·53 | 0·068 |
| U | 0·10 (0·066-0·20)^c^ | 0·16 (0·081-0·36)^c^ | 0·024 (0·013-0·045)^a^ | 0·055 (0·026-0·11)^b^ | <0·001 | <0·001 | 0·34 |

*Data are geometric means (*25^th^-75^th^ percentile*) of urinary concentrations in µg/L*

*^#^ P-values obtained by two-way ANOVA and adjusted with the Benjamini-Hochberg method for multiple testing*

*^a,b,c^ Values with the same letter in superscript do not differ significantly from each other, according to Tukey’s post-hoc test***Table S6.** **Correlation matrix among 24h excreted quantities of urinary metals (µg/day)**

|  | Li | Al | V | Cr | Mn | Co | Ni | Cu | Zn | As | Se | Mo | Cd | Sn | Sb | Te | Ba | Tl | Pb |
| --- | --- | --- | --- | --- | --- | --- | --- | --- | --- | --- | --- | --- | --- | --- | --- | --- | --- | --- | --- |
| Al | 0·46^***^ |  |  |  |  |  |  |  |  |  |  |  |  |  |  |  |  |  |  |
| V | 0·16^*^ | 0·48^***^ |  |  |  |  |  |  |  |  |  |  |  |  |  |  |  |  |  |
| Cr | 0·10 | 0·55^***^ | 0·55^***^ |  |  |  |  |  |  |  |  |  |  |  |  |  |  |  |  |
| Mn | 0·08 | 0·56^***^ | 0·64^***^ | 0·74^***^ |  |  |  |  |  |  |  |  |  |  |  |  |  |  |  |
| Co | 0·26^***^ | 0·63^***^ | 0·54^***^ | 0·72^***^ | 0·66^***^ |  |  |  |  |  |  |  |  |  |  |  |  |  |  |
| Ni | 0·27^***^ | 0·56^***^ | 0·49^***^ | 0·74^***^ | 0·56^***^ | 0·80^***^ |  |  |  |  |  |  |  |  |  |  |  |  |  |
| Cu | -0·10 | 0·40^***^ | 0·36^***^ | 0·61^***^ | 0·56^***^ | 0·58^***^ | 0·45^***^ |  |  |  |  |  |  |  |  |  |  |  |  |
| Zn | 0·06 | 0·32^***^ | 0·18^*^ | 0·45^***^ | 0·36^***^ | 0·45^***^ | 0·40^***^ | 0·51^***^ |  |  |  |  |  |  |  |  |  |  |  |
| As | 0·68^***^ | 0·55^***^ | 0·21^**^ | 0·40^***^ | 0·20^**^ | 0·50^***^ | 0·46^***^ | 0·22^**^ | 0·36^***^ |  |  |  |  |  |  |  |  |  |  |
| Se | 0·63^***^ | 0·47^***^ | 0·14 | 0·35^***^ | 0·13 | 0·37^***^ | 0·42^***^ | 0·17^*^ | 0·27^***^ | 0·75^***^ |  |  |  |  |  |  |  |  |  |
| Mo | 0·67^***^ | 0·24^**^ | 0·12 | 0·05 | 0·04 | 0·33^***^ | 0·32^***^ | -0·04 | 0·04 | 0·44^***^ | 0·50^***^ |  |  |  |  |  |  |  |  |
| Cd | 0·12 | 0·43^***^ | 0·23^**^ | 0·56^***^ | 0·30^***^ | 0·47^***^ | 0·51^***^ | 0·54^***^ | 0·44^***^ | 0·51^***^ | 0·55^***^ | 0·10 |  |  |  |  |  |  |  |
| Sn | -0·02 | 0·35^***^ | 0·14 | 0·61^***^ | 0·30^***^ | 0·50^***^ | 0·51^***^ | 0·55^***^ | 0·43^***^ | 0·37^***^ | 0·36^***^ | -0·08 | 0·69^***^ |  |  |  |  |  |  |
| Sb | 0·39^***^ | 0·57^***^ | 0·24^**^ | 0·63^***^ | 0·43^***^ | 0·58^***^ | 0·53^***^ | 0·44^***^ | 0·33^***^ | 0·59^***^ | 0·61^***^ | 0·24^**^ | 0·56^***^ | 0·60^***^ |  |  |  |  |  |
| Te | 0·32^***^ | 0·22^**^ | 0·13 | 0·32^***^ | 0·15 | 0·34^***^ | 0·34^***^ | 0·12 | 0·15^*^ | 0·48^***^ | 0·67^***^ | 0·35^***^ | 0·54^***^ | 0·36^***^ | 0·48^***^ |  |  |  |  |
| Ba | 0·12 | 0·43^***^ | 0·25^***^ | 0·49^***^ | 0·32^***^ | 0·47^***^ | 0·50^***^ | 0·33^***^ | 0·32^***^ | 0·40^***^ | 0·41^***^ | 0·10 | 0·57^***^ | 0·60^***^ | 0·43^***^ | 0·44^***^ |  |  |  |
| Tl | 0·63^***^ | 0·43^***^ | 0·21^**^ | 0·17^*^ | 0·11 | 0·33^***^ | 0·42^***^ | 0·11 | 0·22^**^ | 0·50^***^ | 0·62^***^ | 0·59^***^ | 0·27^***^ | 0·20^**^ | 0·45^***^ | 0·34^***^ | 0·29^***^ |  |  |
| Pb | 0·06 | 0·38^***^ | 0·41^***^ | 0·49^***^ | 0·56^***^ | 0·52^***^ | 0·46^***^ | 0·65^***^ | 0·33^***^ | 0·19^*^ | 0·11 | 0·14 | 0·25^***^ | 0·42^***^ | 0·50^***^ | 0·03 | 0·24^**^ | 0·38^***^ |  |
| U | 0·39^***^ | 0·74^***^ | 0·47^***^ | 0·54^***^ | 0·43^***^ | 0·59^***^ | 0·55^***^ | 0·37^***^ | 0·30^***^ | 0·52^***^ | 0·46^***^ | 0·17^*^ | 0·43^***^ | 0·37^***^ | 0·65^***^ | 0·20^**^ | 0·42^***^ | 0·35^***^ | 0·32^***^ |

* p<0·05, ** p<0·01, *** p<0·001

Reference List

1. Hoet P, Jacquerye C, Deumer G, Lison D, Haufroid V: **Reference values and upper reference limits for 26 trace elements in the urine of adults living in Belgium.** *Clin Chem Lab Med* 2013, **51:** 839-849.

2. Sughis M, Nawrot TS, Riaz A, Ikram-Dar U, Mahmood A, Haufroid V *et al*.: **Metal exposure in schoolchildren and working children. A urinary biomonitoring study from Lahore, Pakistan.** *Int J Hyg Environ Health* 2014, **217:** 669-677.

3. Paschal DC, Ting BG, Morrow JC, Pirkle JL, Jackson RJ, Sampson EJ *et al*.: **Trace metals in urine of United States residents: reference range concentrations.** *Environ Res* 1998, **76:** 53-59.

4. Komaromy-Hiller G, Ash KO, Costa R, Howerton K: **Comparison of representative ranges based on U.S. patient population and literature reference intervals for urinary trace elements.** *Clin Chim Acta* 2000, **296:** 71-90.
